# Zebrafish caudal fin as a model to investigate the role of probiotics in bone regeneration

**DOI:** 10.1038/s41598-022-12138-z

**Published:** 2022-05-16

**Authors:** Jerry Maria Sojan, Giorgia Gioacchini, Elisabetta Giorgini, Patrick Orlando, Luca Tiano, Francesca Maradonna, Oliana Carnevali

**Affiliations:** 1 Department of Life and Environmental Sciences, Università Politecnica Delle Marche, Via Brecce Bianche, 60131 Ancona, Italy; 2 Biostructures and Biosystems National Institute—Interuniversity Consortium, Viale delle Medaglie d’Oro 305, 00136 Rome, Italy

**Keywords:** Cell biology, Developmental biology, Molecular biology

## Abstract

Probiotics are live microorganisms that confer several beneficial effects to the host, including enhancement of bone mineralization. However, probiotic action on bone regeneration is not well studied and therefore we analysed various effects of probiotic treatment on the caudal fin regeneration of zebrafish. Morphological analysis revealed an increased regenerated area with shorter and thicker lepidotrichia segments after probiotic treatment. Fourier transform infrared spectroscopy imaging analysis highlighted the distribution of phosphate groups in the regenerated fins and probiotic group showed higher amounts of well-crystallized hydroxyapatite. At the midpoint (5 days post amputation) of regeneration, probiotics were able to modulate various stages of osteoblast differentiation as confirmed by the upregulation of some key marker genes such as *runx2b, sp7, col10a1a, spp1* and *bglap*, besides suppressing osteoclast activity as evidenced from the downregulation of *ctsk*. Probiotics also caused an enhanced cell cycle by regulating the expression of genes involved in Retinoic acid (*rarga*, *cyp26b1)* and Wnt/β-catenin (*ctnnb1, ccnd1, axin2, sost*) signaling pathways, and also modulated phosphate homeostasis by increasing the *entpd5a* levels*.* These findings provide new outlooks for the use of probiotics as a prophylactic treatment in accelerating bone regeneration and improving skeletal health in both aquaculture and biomedical fields.

## Introduction

Probiotics are beneficial microbes that can exert numerous health benefits to the host including effects on bone metabolism as reported in various animal models such as poultry and rodents^1–3^. In addition, in the zebrafish model, few studies have provided clear evidence for the role of probiotics in accelerating skeletogenesis and mineralization^4,5^. Probiotic administration basically modulates the host-microbiome^6^ which is proven to affect bone metabolism through many possible ways including metabolite production^7^, hormonal interactive pathways, osteo-immunological responses^8^ or via synthesis of vitamins^9^. Many probiotic species such as *Bacillus subtilis* are known to produce vitamins with osteogenic properties such as vitamin K_2_^10^.

The zebrafish caudal fin has emerged as a highly successful model system for studying the basic mechanisms of tissue regeneration. Under lab conditions, it has several study advantages such as easy live tracking, fin accessibility and the absence of major amputation-causing detrimental effects on the fish^11^. Zebrafish, as a genetic model for bone regeneration, offers the additional worth to translate the findings into bio-medicinal perspective in human regenerative medicines^12,13^. Expression of orthologues for important mammalian osteogenic molecular players like β-catenin has been detected in the regenerating fin in addition to the orthologues for its downstream targets^14–16^. Zebrafish regenerate amputated caudal fins by creating lineage-restricted blastemal cells^17,18^. Following partial amputation, the fin with bony rays and soft inter-ray tissue regenerates very robustly through establishment of blastema, which are populations of lineage-restricted mesenchymal progenitor cells formed via de-differentiation of mature stump cells^19,20^. The pool of de-differentiated osteoblasts in the blastema proliferates, re-differentiates exclusively into non-proliferating osteoblasts and deposit bone matrix during the progression of regeneration^19^. These differentiation steps are distinguished by various osteoblast stage markers such as RUNX family transcription factor 2b (*runx2b*) for osteoblast progenitors towards the distal part of proximal blastema, followed by sp7 transcription factor (*sp7* or osterix) positive osteoblasts and finally bone gamma-carboxyglutamate (gla) protein (*bglap* or osteocalcin) positive mature osteoblasts to the proximal end^21^. Re-expression of *sp7* and the decrease in *bglap* are found to be the indicators of de-differentiation of cells in blastema during regeneration^22^. Several other signaling pathways including RA (Retinoic Acid)^23^ and Wnt/β-catenin^24,25^ have been identified to be essential for fin regeneration.

Based on the evidence from literature, the aim of the present study was to investigate the potential effects of the administration of probiotics on the caudal fin regeneration process. Fish were subject to pre-treatment with probiotics for two weeks before amputation in order to favor colonization of beneficial bacteria in the gut of treated fish and generate desired positive outcomes as previously confirmed with other probiotics species^26^. Using a multidisciplinary approach, ranging from analysis of morphological parameters related to fin growth, to evaluation of expression of representative genes involved in ossification followed by quantification of phosphates and other macromolecules, we aimed at gaining evidence on the role of probiotic bacteria in bone regeneration which could aid in the development of regenerative medicine protocols as well as improve aquaculture practices. In this regard, the possible probiotic action on bone through either increased osteoblast activity or decreased osteoclast activity are both explored in the current study using specific marker genes of osteoblasts and osteoclasts, such as secreted protein, acidic, cysteine-rich (*sparc* or osteonectin) and cathepsin K (*ctsk)*, respectively^27^.

## Results

### Morphometric analysis

**Figure 1:**
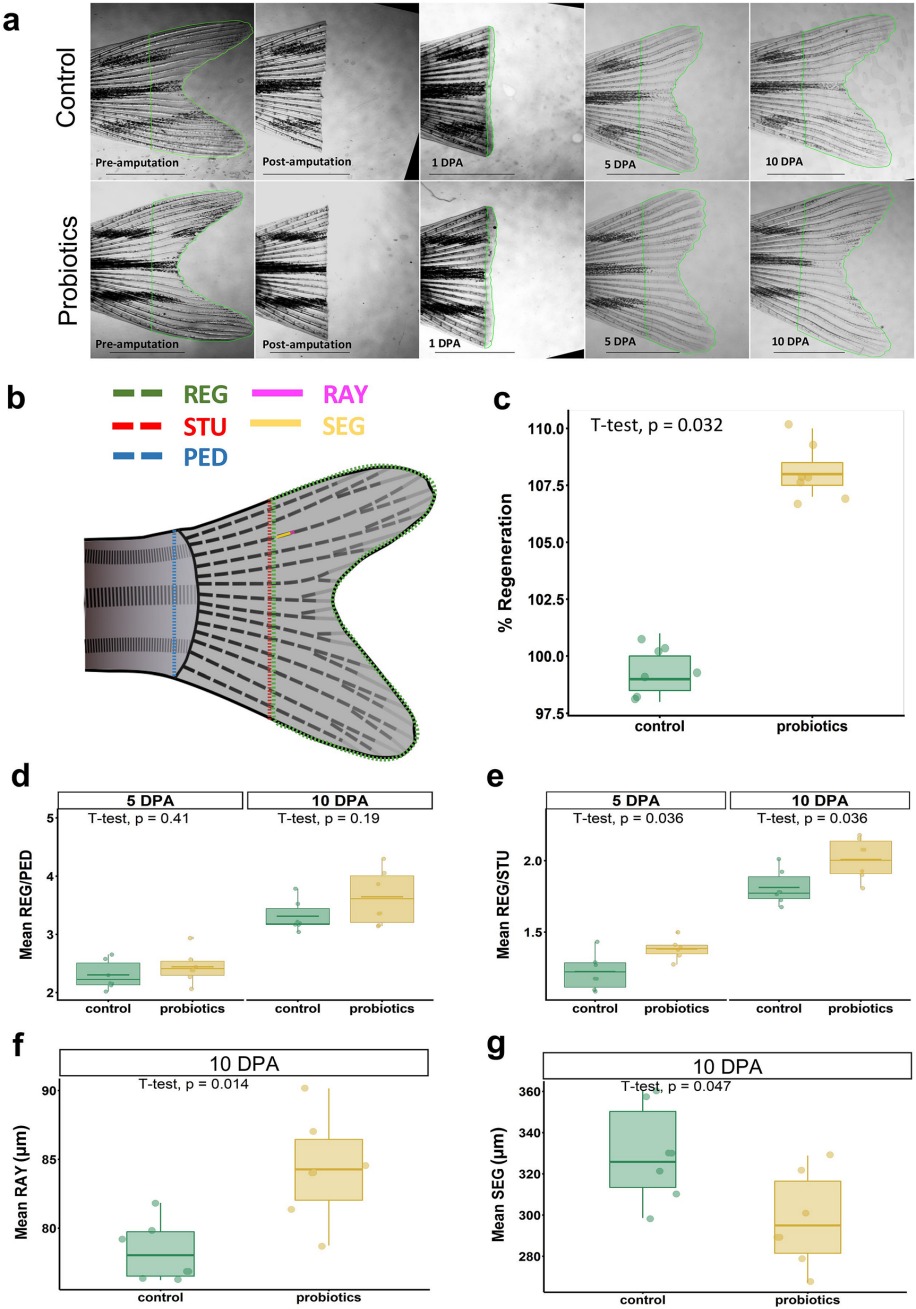
(**a**) Representative photographs showing the fins before amputation, post amputation and at 1, 5 and 10 DPA in control (C) (n = 7) and probiotic-treated (P) (n = 7) groups (Scale bar = 2,000 µm); (**b**) Picture of a representative amputated fin, showing the regenerated area (REG, green dotted tracing), the stump width or width of the amputation plane (STU, red dotted line), the fin ray width (RAY, pink line), the segment length (SEG, yellow line) and the peduncle width (PED, blue dotted line); (**c**) Statistical analysis of regeneration rate (expressed as %) between C and P groups calculated at 10 DPA with respect to the fin before amputation of the same fish. (**d**–**g**) Various morphometric parameters used to analyze the regenerated areas in C and P fins (n = 7 per group per time point) (**d**) Regenerated area (REG)/Peduncle width (PED) ratio; (**e**) Regenerated area (REG)/Stump width (STU) ratio; (**f**) Mean ray width (RAY) and (**g**) Mean segment length (SEG). T-test was used to analyze the differences among the groups. % Values were converted to respective decimal values before applying T-test and statistical significance was set at *p* < 0.05.

To get a preliminary confirmation on whether the probiotic treatment can influence the regeneration process, few morphometric parameters of regenerating fins were analyzed. The regenerative performance between control (C) and probiotic-treated (P) experimental groups was evaluated at 5 days post amputation (DPA) and 10 DPA by analyzing the fin ray width (RAY), calculated by averaging the width of the second bifurcated fin ray in the dorsal lobe at the first formed segment joint after amputation; segment length (SEG), calculated by averaging the length of the first formed segment after amputation in the second bifurcated fin ray in the dorsal lobe; REG/STU, which is the ratio between regenerated area (REG) and stump width (STU), and lastly REG/PED, which is the ratio between REG and peduncle width (PED). The complete course of regeneration was tracked for both C (n = 7) and P (n = 7) fish every day at the same time. In Fig. 1a, images of representative C and P fins from the same tracked fish for pre-amputation, post-amputation and 1, 5 and 10 DPA are reported. A representative image of a regenerating fin with REG, STU, RAY, SEG and PED measurements is shown (Fig. 1b). A specific equation was modified from a previous study to calculate % regeneration by using the ratios REG/STU and Initial amputated area/STU^28^. The modified Eq. (1) is presented below:

1 $$\% Regeneration = \frac{{\frac{REG}{{STU}}\;at\;10\;DPA*100}}{{{\text{Initial}}\;{\text{amputated}}\;{\text{area}}/{\text{STU}}\;{\text{at}}\;0\;{\text{DPA}}}}$$

This parameter was 8–9% higher in P fins than in C group at 10 DPA (Fig. 1c).

REG/STU ratio was significantly higher in P fins than C fins at both 5 DPA (*p* = 0.036) and 10 DPA (*p* = 0.036) whereas no significant difference was observed between the groups for REG/PED ratio even though it displayed the same pattern as that of REG/STU (Fig. 1d,e). Stump width (STU) was used to normalize the inter-specimen variability arising due to variable size and alignment of fin; therefore REG/STU is considered as the best standard to normalize the regenerated area^29^. Hence, the lack of significant difference for the ratio REG/PED between P and C could be due to a non-significant increase in PED linked to a possible inter-specimen variability in body size. Another interesting observation was that P group had thicker fin rays (Mean RAY; Fig. 1f) and shorter segments (Mean SEG; Fig. 1g) with respect to C at 10 DPA. No significant differences in body weight and total length were observed between C and P fish during the trial. These results suggest that P treatment accelerated the regeneration process and the larger regenerated P fins had shorter but thicker segments.

### FTIRI (Fourier transform infrared spectroscopic imaging) analysis

**Figure 2:**
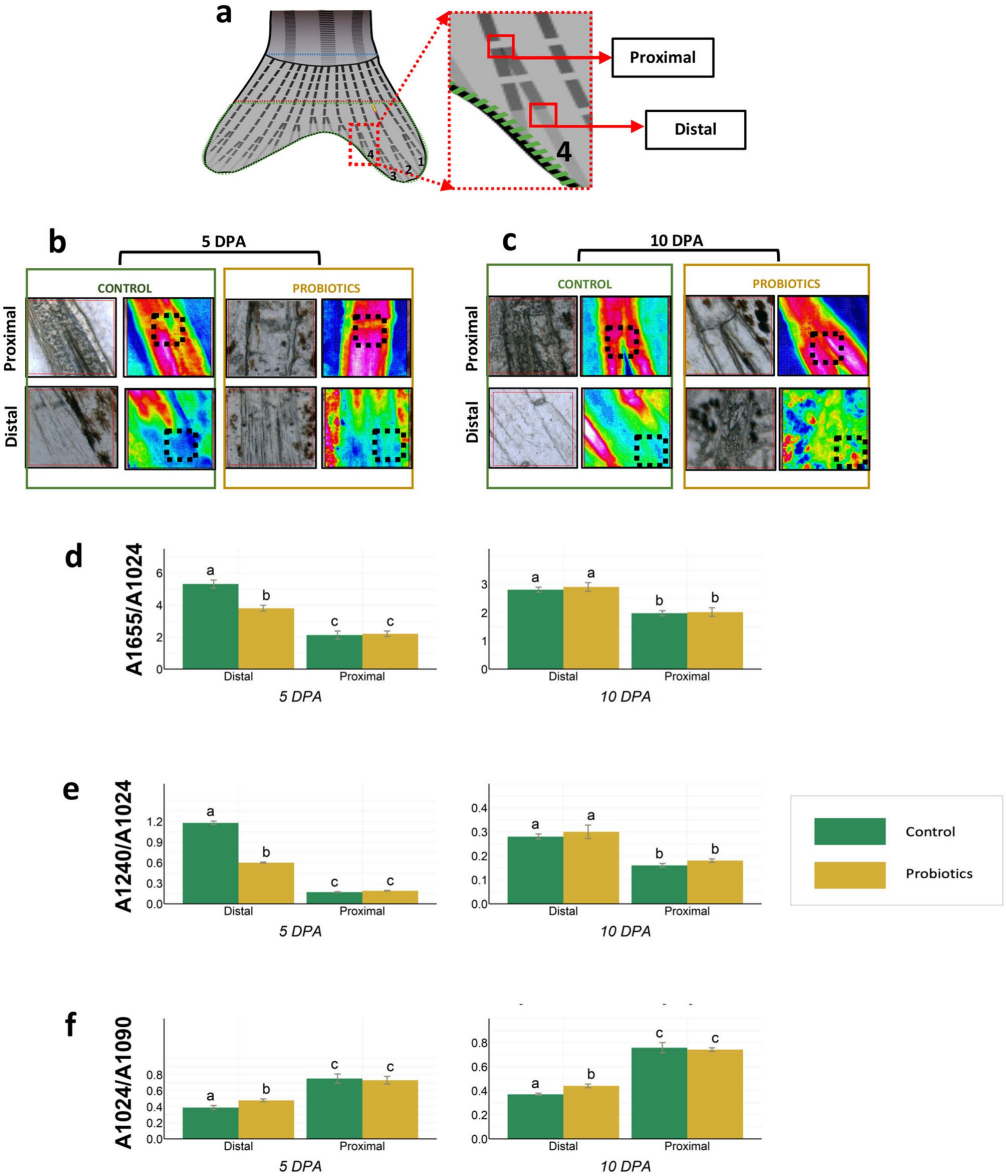
(**a**) Representative picture showing the areas analyzed by FTIRI in the fourth bifurcated fin ray of the dorsal lobe of the regenerating fin. The two regions assayed after amputation were: proximal-corresponding to the first formed bifurcation after amputation and distal-corresponding to the last segment joint preceding the actinotrichia; (**b** and **c**) Representative microphotograph (left) and false color images (right) denoting the topographical distribution of phosphate groups in the proximal and distal areas of the regenerated fin from C and P fins (n = 3) at (**b**) 5 DPA and (**c**) 10 DPA time points. The same color scale (0–3) was used for all false color images: white/light pink colors indicate the areas with the highest amount of phosphates, red/orange/yellow indicate the areas with an intermediate amount and black/dark blue shows the areas with the lowest amount of phosphates. The black dotted squares indicate the region where spectra were extracted for the curve fitting analysis; (**d**–**f**) Biochemical composition and bone mineralization assessed by FTIRI analysis.  Histograms representing band area ratios (**d**) A_1655_/A_1024_, (**e**) A_1240_/A_1024_ and (**f**) A_1024_/A_1090_ are calculated on proximal and distal regions of the regenerating fin from C (n = 3) and P (n = 3) fins at 5 DPA and 10 DPA. Data are presented as mean ± S.D. Different letters over the histograms indicate statistically significant difference among groups. Two-way ANOVA and Tukey’s multiple comparison test are used, and statistical significance was set at *p* < 0.05.

FTIRI is a suitable tool to study bone composition, particularly the mineral and organic matrix content by calculating relative concentrations using peak intensity ratios of various chemical components^30^. In Fig. 2a, a representative image of the fin showing the two regions of FTIRI analysis, proximal and distal, on the fourth bifurcated fin ray in the dorsal fin lobe is presented. In Fig. 2b,c, the hyperspectral imaging analysis of C and P amputated fins are reported at two time points of regeneration, 5 and 10 DPA. As expected, a non-homogeneous distribution was observed within the mapped areas and the distribution of phosphates clearly matched with bone structures in both the regions analysed. In the actinotrichia (see black dotted square in the distal maps; Fig. 2b,c), a lower phosphate level was detected indicating a lower mineralization of this zone.

To evaluate the biochemical composition and degree of mineralization of bone in the two analyzed regions, the following band area ratios were calculated: A_1655_/A_1024_ (ratio between the area of the Amide I band of proteins centered at 1655 cm^−1^ and the area of the phosphate band centered at 1024 cm^−1^; Fig. 2d); A_1240_/A_1024_ (ratio between the area of the collagen band centered at 1240 cm^−1^ and the area of the phosphate band centered at 1024 cm^−1^; Fig. 2e) and A_1024_/A_1090_ (ratio between the area of the phosphate band centered at 1024 cm^−1^ and 1090 cm^−1^, ascribable respectively to well and poorly crystallized hydroxyapatites (HA); Fig. 2f). The A_1655_/A_1024_ and A_1240_/A_1024_ ratios are usually related to the relative amount of the organic component (proteins and collagens) with respect to the inorganic/mineral one (bone HA)^31^. These two ratios showed a decrease in the proximal area with respect to distal one. Moreover, at 5 DPA, in the distal region, statistically significant lower values were found for these ratios in P group respect to C (*p* < 0.05), indicative of a higher amount of the mineral component in the P fins. Conversely, no statistically significant difference was observed for these two ratios at 10 DPA between C and P groups in both analyzed regions (*p* > 0.05) (Fig. 2d,e). The A_1024_/A_1090_ ratio is referred to the mineral maturity of bone, representing the transformation of HA from a nanocrystalline form (represented by the peak at 1090 cm^−1^) to a well-crystallized stoichiometric one (represented by the peak at 1024 cm^−1^)^32^. In the distal region, statistically significant higher values were found in P samples at both 5 DPA and 10 DPA with respect to C. In the proximal region, the ratio remained same between C and P samples (*p* > 0.05) (Fig. 2f). Altogether, these results suggest an increase of mineral content and mineral maturity in the regenerated fins due to P treatment.

### Marker genes analysis by real time PCR (qRT-PCR)

#### Expression of early, intermediate, and late markers of osteoblast differentiation

**Figure 3:**
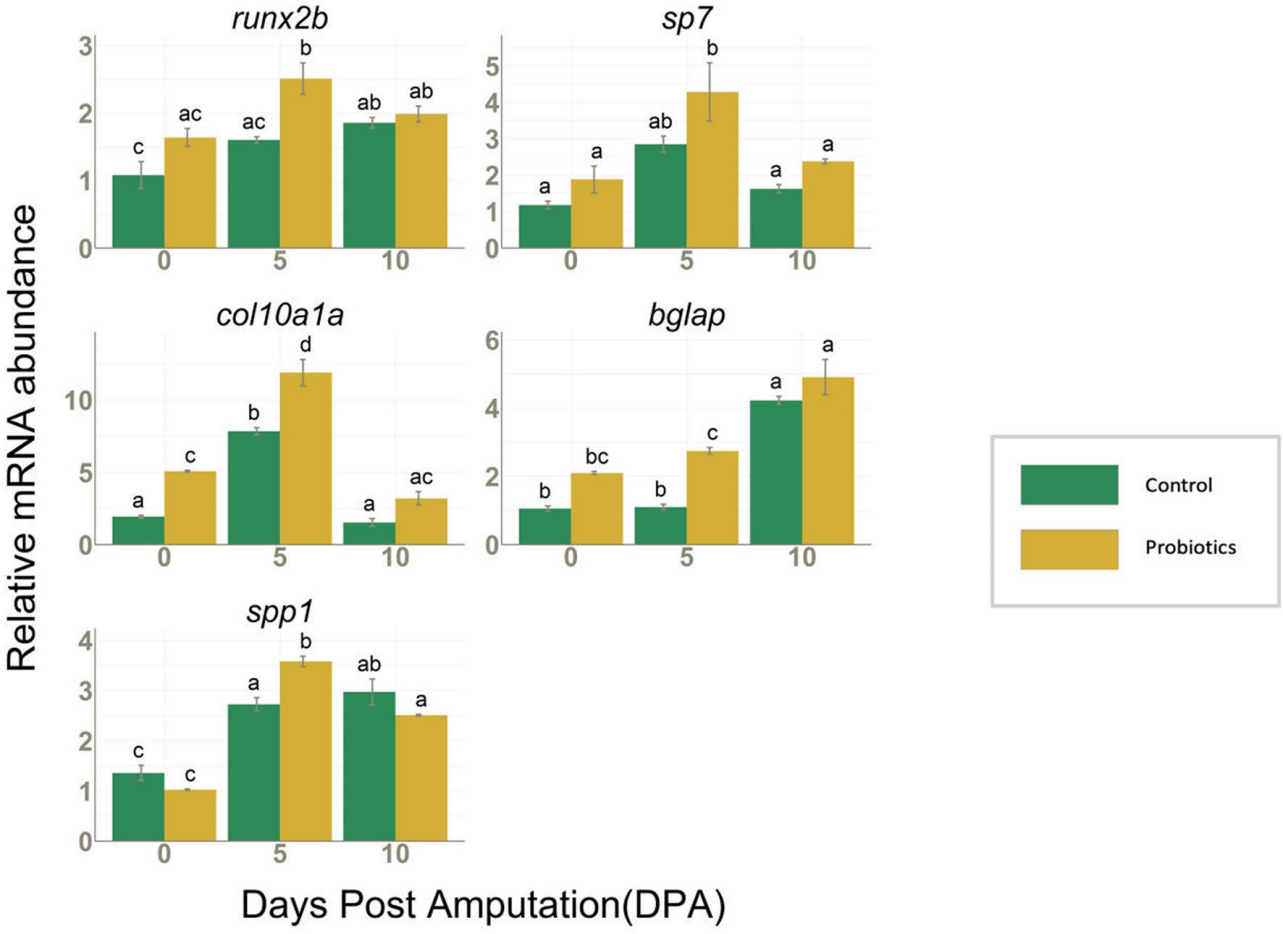
*runx2b, sp7, col10a1a, bglap* and *spp1* mRNA values normalized against *rplp0* and *rpl13* in fins collected from C (n = 3) and P (n = 3) groups at 0, 5 and 10 DPA. Data are presented as mean ± S.D. Different letters over the histograms indicate statistically significant difference among groups. Two-way ANOVA and Tukey’s multiple comparison test are used, and statistical significance was set at *p* < 0.05.

Fin regeneration is a process where cells ranging from pre-osteoblast to mature osteoblast are involved. Therefore, it is essential to consider that probiotic treatment can act differently on various osteoblast stages. In our study, mRNA levels of early and intermediate markers of osteoblast differentiation such as *runx2b, sp7* and collagen 10a1a (*col10a1a)* increased during the regeneration at 5 DPA with respected to 0 DPA in both C and P. Both *runx2b* and *col10a1a* mRNA showed a significantly higher expression in P group with respect to C at 5 DPA and *col10a1a* was also highly expressed in the P fins at 0 DPA. The late marker *bglap* was significantly increased in both C and P when nearing the completion of the regeneration process (10 DPA) but a significant difference between C and P was observed particularly at 5 DPA. The expression of mature osteoblast specific marker, secreted phosphoprotein 1 (*spp1* or osteopontin), mRNA was significantly higher in treated fins at 5 DPA (Fig. 3). The results indicate that P treatment can boost osteoblast advancement from early differentiation to extracellular matrix mineralization.

#### Expression of genes associated with the regulation of RA signaling and phosphate homeostasis during regeneration

**Figure 4:**
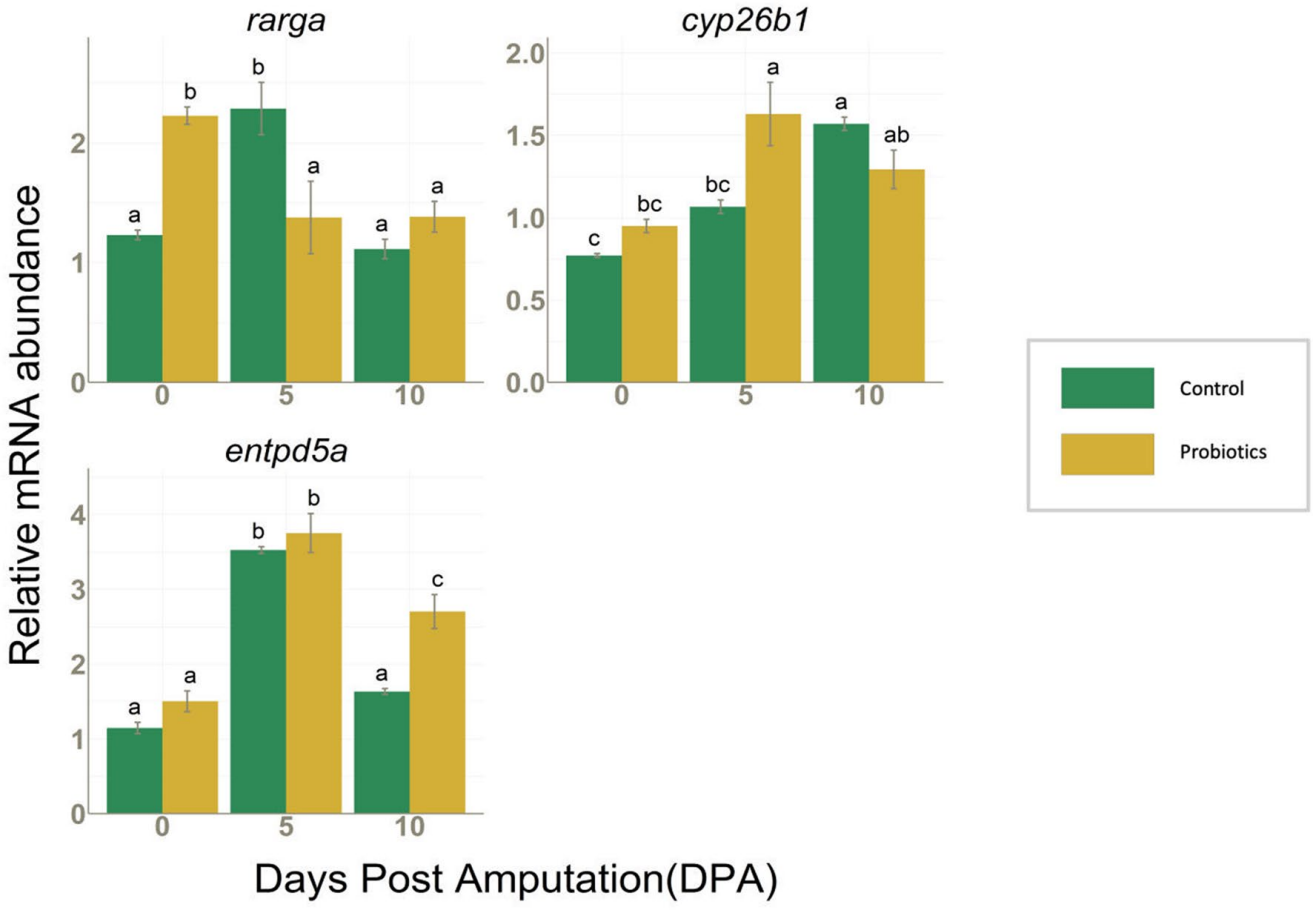
*rarga, cyp26b1* and *entpd5a* mRNA values normalized against *rplp0* and *rpl13* in fins collected from C (n = 3) and P (n = 3) groups at 0, 5 and 10 DPA. Data are presented as mean ± S.D. Different letters over histograms indicate statistically significant difference among groups. Two-way ANOVA and Tukey’s multiple comparison test are used, and statistical significance was set at *p* < 0.05.

Previous studies reported the RA signaling involvement in the blastemal cell proliferation, a key process in fin regeneration which is also regulated by Wnt/β-catenin signaling^33^. Furthermore, skeletogenesis in zebrafish requires a precise control of RA levels and Cyp26b1 activity^34^. At 5 DPA, the RA degrading enzyme, codified by cytochrome P450, family 26, subfamily b, polypeptide 1 (*cyp26b1*) mRNA*,* was significantly higher in P group and was associated to a decreased expression of retinoic acid receptor gamma-a (*rarga*) with respect to C. *Rarga* mRNA expression was also found to be higher in the initial amputated fins (0 DPA) of P treated group than in C. An increase in the expression of ectonucleoside triphosphate diphosphohydrolase 5a (*entpd5a*), which is involved in the homeostasis of phosphates, was also observed from 0 to 5 DPA in both C and P groups. At 10 DPA, the *entpd5a* gene expression declined and only at this time point, its mRNA level in P group was significantly higher with respect to the control (Fig. 4). The expression changes observed across these genes indicate that probiotic treatment can modulate the RA signaling pathway and also regulate phosphate homeostasis during regeneration.

#### Expression of genes associated with the regulation of Wnt/β-catenin signaling during regeneration

**Figure 5:**
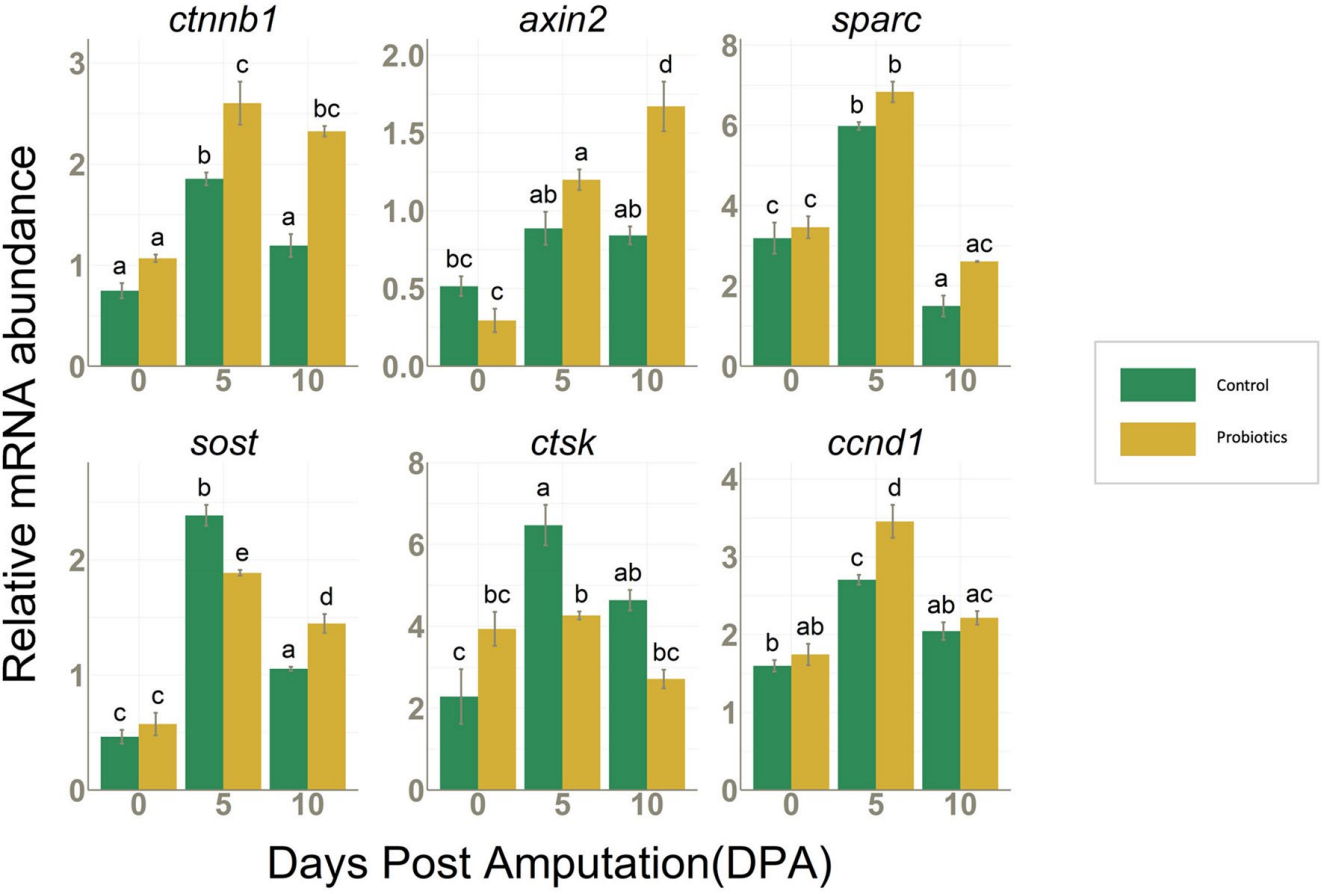
*ctnnb1, axin2, sparc, sost, ctsk* and *ccnd1* mRNA values normalized against *rplp0* and *rpl13* in fins collected from C (n = 3) and P (n = 3) groups at 0, 5 and 10 DPA. Data are presented as mean ± S.D. Different letters over histograms indicate statistically significant difference among groups. Two-way ANOVA and Tukey’s multiple comparison test are used, and statistical significance was set at *p* < 0.05.

Wnt/β-catenin mediated signaling plays an important role in blastema cell proliferation^16^ and analyzing the expression of some key genes involved in this pathway can provide essential information on the effect of probiotic treatment on proliferation and early differentiation of osteoblasts during fin regeneration. The catenin (cadherin-associated protein), beta 1(*ctnnb1*) was significantly higher in P group at both 5 DPA and 10 DPA whereas its universal transcriptional target gene *axin2,* which is also a negative feedback regulator in the canonical Wnt signaling, was significantly higher in P fins at 10 DPA*. Sparc,* representing the gene encoding for sparc protein, which are known to prevent the degradation of β-catenin, was also significantly higher at 5 DPA in both groups with respect to 0 DPA but without any significant difference between C and P groups. The negative regulator of Wnt signaling, sclerostin (*sost*), and the osteoclast marker gene *ctsk* were significantly higher in C at 5 DPA whereas at 10 DPA, *sost* was higher in P group than in C. Another downstream transcriptional target gene of β-catenin is cyclin D1(*ccnd1*), which is also a marker of cell proliferation, and its mRNA was also upregulated in P fins with respect to C at 5 DPA (Fig. 5). Altogether, these results provide evidence that the increase in β-catenin transcription activity drives the expression of several downstream regulator genes which are also involved in fin regeneration process. Thus, Wnt/β-catenin signaling pathway was found to be modulated by P treatment leading to an enhanced cell cycle, thereby accelerating the regeneration process, and led to an increased regenerated area as evidenced by the morphological results.

## Discussion

Using the established zebrafish fin regeneration model, we evaluated the modulation of regeneration in zebrafish caudal fin by probiotic treatment. Since treated fish showed an increased area of regenerated fin compared to the initial amputated area but not changes in total length, weight and peduncle width, the increased regenerated area can be conclusively linked to the effect of P treatment on regeneration and not to fish growth or fin alignment. In addition, C fins switched back to isometric growth once the amputated area is regenerated whereas the P fins maintained the allometric growth pattern of regeneration even after reaching the original amputated area. This led to a significantly increased outgrowth of fin area with shorter but thicker segments of fin rays than in C. Previous studies have described a similar outgrowth during fin regeneration by inhibiting the protein phosphatase calcineurin and also by an integrated effect of calcineurin inhibition and bioelectric signaling like potassium channels^35,36^. Thus, we can speculate that P treatment could play a role in the activation of the above mentioned signaling causing a delay to the conventional switching back of the fin growth to isometric pattern.

Calcineurin-inhibition related fin outgrowth was previously reported to be also associated with the promotion of RA signaling^35,37^ and therefore, in this study, a possible modulation of RA signaling by P in the regenerated fins was taken into consideration. In addition, RA signaling is also known to affect the differentiation and mineralization of osteoblasts along with proliferation^34^, and this suggested us to explore RA signaling in the light that other osteoblast differentiation marker genes were also affected by the P treatment (Fig. 3). In the initial amputated fins (0 DPA) which received the probiotics preconditioning for 14 days, interestingly an upregulation in the RA receptor, *rarga* as well as *col10a1a* was observed. Indeed, the positive effects of P exposure on RA pathway were previously described in probiotic treated zebrafish favoring an enhanced calcification of vertebrae^4^. This verifies the activity of probiotics in regulating genes with a role in the bone calcification besides modulating regeneration process. During the regeneration, *rarga,* which is expressed during the blastema formation^38^, increased in C fins accordance with the established molecular regulation of regeneration process but a decrease was noted in P fins at 5 DPA. Considering that *cyp26b1*-mediated RA degradation plays an important role in promoting the re-differentiation of the pre-osteoblasts to the non-proliferative osteoblasts which is an essential requirement for the formation of new bones^23^, the higher RA levels in C fins respect to P fins, suggests the promotion of the proliferation of the pre-osteoblasts and less re-differentiation whereas P fins are at a relatively more advanced stage of regeneration involving re-differentiation. Moreover, higher levels of *entpd5a* in the completely regenerated P fin suggests a higher phosphate concentration, since *entpd5a* is a direct regulator of phosphate homeostasis^39^. Nevertheless, at both time point analyzed, FTIR results show that in the distal zone, P fins present higher amount of phosphates as crystallized HA. Since this part of fin, according to the physiology of bone regeneration is the newly formed, these results let us speculate that P treatment accelerate the transformation of phosphates from amorphous to a more organized form, thereby boosting the bone maturation process^40^.

The significantly higher expression of early and intermediate osteoblast differentiation stage markers such as *runx2b, sp7, ctnnb1* and *col10a1a* in P fins at the mid-point of regeneration (5 DPA) confirms the role of probiotics in modulating the regeneration process at various stages of osteoblast de-differentiation and proliferation. *Runx2b* is a pre-osteoblast stage marker, which expresses in the blastema during the de-differentiation of osteoblasts in the early stages of regeneration^21^ and *sp7* acts hierarchically downstream to the *runx2b* in the differentiation pathway. *Runx2b* also regulates the expression of other important marker genes of various stages of osteoblast differentiation like *col10a1a, spp1, bglap* and hence is considered as a key regulator. *Col10a1a*, which acts further downstream to *sp7*, marks the intermediate stages of skeletogenesis (bone matrix deposition stages)^41^. It is expressed in early osteoblasts and chondrocytes during intramembranous and perichondral ossification of fish bone^42–45^ and our results showed its strong downregulation towards the final stage of regeneration (10 DPA). The sequential activation of early markers points to the same conclusion that P treatment significantly promotes the early and intermediate stages of osteoblast differentiation processes after fin amputation. Regarding mineralization, established marker genes of later stages of osteoblast differentiation and extracellular matrix mineralization such as *bglap* (osteocalcin) and *spp1* (osteopontin), both acting downstream and regulated by *sp7* and *runx2,* were evaluated^21,22,46^. In our study, both were upregulated during the regeneration process with a significantly higher expression in the treated fins at 5 DPA. These results are further supported by the higher crystallized HA levels found in P fins, providing clear evidence of the effect of P on the mineralization stage as well.

*Ctnnb1* (β-catenin) transcript was significantly higher in P fins during regeneration suggesting an activation of Wnt/β-catenin signaling by P. From the expression of two downstream target genes of β-catenin, cell proliferation marker *ccnd1* and negative regulator of β-catenin signaling *axin2,* we could observe that the increased β-catenin signaling at 5 DPA was suppressed towards 10 DPA in both the groups. This confirms that the measured levels of β-catenin are well related with the different regeneration stages, being higher during proliferative phase and lower during the later stages of differentiation/maturation. Elevated expression of *sparc* at 5 DPA further confirms the previous reports of the involvement of this pathway in the fin regeneration as *sparc* codes for a matrix protein which is known to increase osteoblastogenesis through enhancing β-catenin mediated signaling and preventing the degradation of β-catenin^47^. *Sparc* was earlier found to be enriched at 4 DPA regenerates^27^ which further agrees with our observation of significantly higher amount of *sparc* at 5 DPA, although no differences were observed between groups. Also, the higher expression of *sost* at the midpoint of regeneration is an additional indicator for the modulation of Wnt/β-catenin signaling during blastema formation, since sclerostin is found to be expressed in the blastema during early fin regeneration in zebrafish^16^. In our study, P caused the downregulation of *sost* mRNA levels in P fins and a previous trial on zebrafish larvae also reported regulation of *sost* levels by probiotic treatment^5^. Wnt/β-catenin signaling is also involved in the suppression of osteoclast activity^48,49^ and this evidence is strongly supported by our results showing P ability to downregulate the osteoclast marker gene *ctsk*^27^ during regeneration.

The overall results suggest that P treatment positively affects the caudal fin regeneration process. At molecular level, P mainly affected the regeneration at midpoint (summarized in Supplementary Fig. S3) and the major pathways modulated are Wnt/β-catenin and RA signaling. The treatment induced an increased regenerated area, affected the morphology of the fins, and enhanced well-crystallized HA content. Furthermore, FTIRI can be proposed as a suitable tool to investigate on regeneration process. Since probiotics can have their regulatory effects through multiple ways, studies investigating the exact mode of action of probiotics on regeneration at cellular level could be done in the future as a follow-up of these positive results. Since this probiotic mix contains major vitamin K_2_ producers such as *Bacillus subtilis* and vitamin K_2_ is a crucial class of vitamins known to partake in the positive regulation of bone development^50^, further studies can be done focusing on probiotic ability to produce vitamin K_2_. In brief, the significant impact of probiotic treatment on the regeneration process was revealed using zebrafish caudal fin as a model. This observation could be useful in bone regenerative medicine studies where probiotics can be a potential prophylactic candidate to improve bone health.

## Methods

### Ethics declarations and approval for animal experiments

All procedures involving animals were conducted in accordance with the Italian law on animal experimentation and were approved by the Ethics Committee of the Università Politecnica delle Marche, Ancona, Italy and by The Italian Ministry of Health (Aut. No. 583/2020-PR). All efforts were made to minimize animal suffering and the study was carried out in compliance with the ARRIVE guidelines.

### Probiotics administration and caudal fin regeneration

Three months old wild-type (AB) zebrafish with a mean weight of 100 ± 7 mg and a mean total length of 20 ± 2 mm, maintained at 28.0 °C, pH 7.0, photoperiod 12:12 light: dark, NO_2_ < 0.01 mg/L and NO_3_ < 10 mg/L, were collected from the fish facility and divided into a control group (C) (n = 24) and a probiotic-treated group (P) (n = 24). The trial was conducted using a commercial probiotic mixture, Bactosafe H (Bernaqua), which consists of a mix of 5 different bacteria- *Bacillus subtilis, Bacillus licheniformis, Bacillus coagulans and Lactobacillus acidophilus* plus the yeast *Saccharomyces cerevisiae*. Within the probiotic mix, *B.subtilis*, the strain of our interest is known to produce vitamin K_2_ or menaquinones, a group of pro-osteogenic vitamins^10^. C and P groups were fed a commercial diet (Zebrafeed, Sparos, Portugal) at 3% body weight twice a day and only P group received a dietary supplementation with the probiotics at 10^6^ CFU/ml. A 14-day pre-conditioning treatment with the probiotics was administered to the P group to enhance gut colonization. At the end of pre-conditioning, fish were anesthetized using 0.1 g/l MS-222 (Sigma-Aldrich, USA) and the caudal fins were amputated 1–2 segments anterior to the bifurcation of the second bifurcated lepidotrichia^29^. Amputated fins (0 DPA) were stored at -80 °C for RNA extraction. After amputation, each fish was maintained separately, to have biological replicates at a density of 1 fish per 200 ml in glass containers filled with water from the rearing system. The glass containers were maintained in a water bath equipped with heaters and air stones to keep the temperature homogenous. Fish were allowed to regenerate at 33.0 (± 1) °C to accelerate regeneration as previously reported^51^. During the regeneration, fish were fed with the same quantity of commercial feed and administered with the same P concentration, as in the 14 days of pre-conditioning. Individual regeneration process was tracked (n = 7 per group) by taking images of the same fish at pre-amputation, post amputation and 1, 5 and 10 DPA. Regenerated caudal fin samples were collected at 5 DPA, representing the mid time point of regeneration, and at 10 DPA as the last point of regeneration for RNA extraction and FTIRI analysis. Fins for RNA extraction (n = 9 per group per time point) were stored at -80 °C. For FTIRI analysis, the fins (n = 3 per group per time point) were fixed in 4% paraformaldehyde (PFA) for 12 h, washed twice with PBS and then stored in PBS at + 4 °C.

### Microscopy and image analysis

Images were taken using a stereomicroscope (Leica, Germany) at pre-amputation, post amputation and 1, 5 and 10 DPA for tracking the progress of regeneration in C and P fins (n = 7 per group). Fish were anaesthetized before the imaging using MS-222 at 0.6 mM and after the imaging, fish could recover in fresh water with aeration. Morphological studies were performed by measuring some of the previously described parameters: REG (regenerated area), PED (peduncle width), STU (stump width), RAY (fin ray width) and SEG (segment length) and two ratios REG/STU and REG/PED^29^. Fin images were analyzed using ImageJ (version 2.1.0/1.53c; Wayne Rasband, National Institutes of Health, USA) and ImageJ macros used to analyze the images was scripted specifically for the regeneration parameters of caudal fin (provided as Supplementary Data S1).

### FTIRI analysis

FTIRI analysis was performed by a Bruker INVENIO interferometer coupled with a Hyperion 3000 IR-Vis microscope and equipped with a FPA detector (Bruker Optics, Ettlingen, Germany). Fin samples from C and P experimental groups (n = 3 per group per time point) were deposited onto CaF2 optical windows. By using a 15× condenser objective, specific areas were selected on each fin sample. The two regions assayed after amputation in the fourth bifurcated fin ray of the dorsal fin lobe are described as: proximal- corresponding to the first distal bifurcation and distal- corresponding to the last segment joint preceding the actinotrichia (see Fig. 2a). On these areas, the IR maps were acquired in transmission mode in the 4000–800 cm^−1^ spectral range. Each map was 164 × 164-micron side, and it was the result of 4096 pixel/spectra (256 scans), with a spatial resolution of 2.56 × 2.56 micron. Raw IR maps were corrected for carbon dioxide and water vapour and then vector normalized in the full spectral range (Atmospheric Compensation and Vector Normalization routines, OPUS 7.5 software package). False colour images representing the topographical distribution of phosphate groups were obtained by integrating all IR maps in the 1185–980 cm^−1^ spectral range (assigned to the stretching vibrations of phosphate groups). An arbitrary color scale was used where white/light pink colours represent the zones with the highest absorption of phosphates whereas black/dark blue denote the zones with the lowest absorption of phosphates.

On each IR map, a submap (ca. 300 spectra/pixel) was extracted in correspondence with the bone at the two analyzed regions. The average spectrum and average ± standard deviation spectra were calculated, and curve was fitted in the 1800–900 cm^−1^ spectral range. The number and the position (expressed in wavenumbers, cm^−1^) of the underlying bands were identified by second derivative minima analysis and fixed during procedure with Gaussian functions (GRAMS/AI 9.1, Galactic Industries, Inc., Salem, New Hampshire). The integrated areas (A) of the underlying bands were used to calculate the following band area ratios: A_1655_/A_1024_ (Protein to Phosphate ratio), A_1240_/A_1024_ (Collagen to Phosphate ratio), and A_1024_/A_1090_ (Well crystallized HA to Poorly crystallized HA ratio).

### RNA extraction and quantification

Pools of 3 fins were made to have 3 replicates per group for each time point. Total RNA was extracted from amputated caudal fin (0 DPA) and regenerated caudal fins (5 and 10 DPA) using RNAeasy Microkit (Qiagen, Italy). It was then eluted in 20 µl of molecular grade nuclease free water. Final RNA concentrations were determined using a nanophotometer. Total RNA was treated with DNase (10 IU at 37 °C for 10 min, MBI Fermentas). One microgram of total RNA was used for cDNA synthesis using iScript cDNA Synthesis Kit (Bio-Rad, Italy) and stored at -20 °C until further use as described previously^52^.

### qRT-PCR

qRT-PCRs were performed in C and P fins (n = 3 for both C and P, at each time point) with SYBR green (Bio-Rad, Milan, Italy) in a CFX thermal cycler (Bio-Rad, Milan, Italy) as described before^53^. The thermal profile for all reactions was 3 min at 95 °C followed by 45 cycles of 20 s at 95 °C, 20 s at 60 °C and 20 s at 72 °C. Dissociation curve analysis showed a single peak in all the cases. Ribosomal protein L13 (*rpl13*) and ribosomal protein, large, P0 (*rplp0*) were used as the housekeeping genes to standardize the results by eliminating variation in mRNA and cDNA quantity. No amplification product was observed in negative controls and primer-dimer formation was never seen. Data was analyzed using iQ5 Optical System version 2.1 (Bio-Rad) including Genex Macro iQ5 Conversion and Genex Macro iQ5 files. Modification of gene expression between the experimental groups is reported as relative mRNA abundance (Arbitrary Units). Primers were used at a final concentration of 10 pmol/ml. All primer sequences used in the study are listed in Supplementary Table S2.

### Statistical analysis

T-test was used to analyze the morphological regeneration parameter differences between the groups. Two-way analysis of variance (ANOVA) followed by Tukey’s post hoc tests were applied to both IR and qRT-PCR data, to compare differences among experimental groups. All the tests were performed using R version 3.6.1^54^ and plots were generated using ggplot2 3.2.1. Statistical significance was set at *p* < 0.05 for all the tests.

## Supplementary Information

Supplementary Information.

## Data Availability

All data generated or analysed during this study are included in this published article and its Supplementary Information files.
