# Supplementary material for: Zebrafish caudal fin as a model to investigate the role of probiotics in bone regeneration
Source: Sci Rep. 2022 May 16;12:8057. doi: 10.1038/s41598-022-12138-z (PMC9110718; doi:10.1038/s41598-022-12138-z)
Supplement: Supplementary file 1 — Supplementary Information. [file 41598_2022_12138_MOESM1_ESM.docx]

SUPPLEMENTARY INFORMATION

**Zebrafish caudal fin as a model to investigate the role of probiotics in bone regeneration**

Jerry Maria Sojan^1^, Giorgia Gioacchini^1^, Elisabetta Giorgini^1^, Patrick Orlando^1^, Luca Tiano^1^, Francesca Maradonna ^1,2, *^ and Oliana Carnevali ^1,2,^ ^*^

**Supplementary Data S1**. Script of ImageJ macros used to analyze the regeneration parameters in C and P caudal fins

Macro "Fin regeneration”

D17D23D27D2bD33D34D37D3aD3bD40D41D42D43D44D45D47D49D4aD4bD4cD4dD4eD53D54D57D5aD5bD63D67D6bD77D83D87D8bD93D94D97D9aD9" {

//select your output folder

Dialog.create("Output folder");

Dialog.addMessage("Select the output directory");

Dialog.show();

G_Ddir = getDirectory("Destination Directory to save results");

//select your input folder

Dialog.create("Input folder");

Dialog.addMessage("Select the Input directory");

Dialog.show();

G_Sdir = getDirectory("Directory of raw data");

list = getFileList(G_Sdir);

print("image_title" + "\t" + "PED" + "\t" + "STU" + "\t" + "REG" + "\t" + "RAY" + "\t" + "SEG");

//loop through images

for(i = 0; i<list.length; i++) {

Analysis_func(list[i]);

}

selectWindow("Log");

//save your final output as excel

saveAs("Text", G_Ddir + "test1.csv" );

close("Log")

}

function Analysis_func(image_filename) {

fullpath_image = G_Sdir + image_filename;

open(fullpath_image);

imageID = getImageID();

image_title = getTitle();

close("B&C");

//straight line to measure peduncle width

setTool("line");

waitForUser("Measure peduncle width");

getStatistics(Length);

run("Flatten");

y1 = Length;

selectImage(imageID);

close();

//Measure the STU

imageID2 = getImageID();

setTool("polyline");

waitForUser("Measure stump width");

getStatistics(Length);

run("Flatten");

y2 = Length;

selectImage(imageID2);

close();

//REG area calculation

imageID3 = getImageID();

setTool("polygon");

waitForUser("Trace around the regenerated area");

getStatistics(area);

run("Flatten");

y3 = area;

selectImage(imageID3);

close();

//RAY WIDTH

imageID4 = getImageID();

setTool("polyline");

waitForUser("measure ray width");

getStatistics(Length);

run("Flatten");

y4 = Length;

selectImage(imageID4);

close();

// mesure SEGMENT LENGTH

imageID8 = getImageID();

setTool("polyline");

waitForUser("Measure segment length");

getStatistics(Length);

run("Flatten");

y5 = Length;

selectImage(imageID8);

close();

//print results into log window

print(image_title + "\t" + y1 + "\t" + y2 + "\t" + y3 + "\t" + y4 + "\t" + y5);

//move original image

File.rename(fullpath_image, G_Ddir + image_title);

selectImage(imageID19);

file_rename2 = image_title + "_final.tif";

fullpath = G_Ddir + file_rename2;

saveAs("tiff", fullpath);

close();

close();

}

**Supplementary Table S2**. List of primers used in the qRT-PCR

| **Gene Acronym** | **NCBI gene accession no** | **Forward** | **Reverse** |
| --- | --- | --- | --- |
| ***col10a1a*** | NM_001083827.1 | CCCATCCACATCACATCAAA | GCGTGCATTTCTCAGAACAA |
| ***runx2b*** | NM_212862.2 | GTGGCCACTTACCACAGAGC | TCGGAGAGTCATCCAGCTT |
| ***spp1*** | NM_001002308.1 | GAGCCTACACAGACCACGCCAACAG | GGTAGCCCAAACTGTCTCCCCG |
| ***cyp26b1*** | NM_212666.1 | GCTGTCAACCAGAACATTCCC | GGTTCTGATTGGAGTCGAGGC |
| ***rarga*** | NM_131339.1 | ATTCCGCCAGAGAGCTATGA | TAGGCCCAGGTCTAGCTGAA |
| ***ctnnb1*** | XM_005157831.4 | CGCACACATTCACTCTCAGC | TGGGTAGCCATGATTTTCTCA |
| ***entpd5a*** | XM_679770.8 | ATATGCCTGAAAAGGGTGGA | TACTTCTTTGACCTCATTCAGCAG |
| ***ctsk*** | NM_001017778.1 | GATGAGGCTTGGGAGAGCTGGAA | TTTCGGTTACGAGCCATCAGGAC |
| ***sparc*** | NM_001001942.1 | TGCTTAGGCTGAAACTCAAGATGAG | GCATCAATGGAAGACGTCCTTAGAT |
| ***sp7*** | NM_212863.2 | AACCCAAGCCCGTCCCGACA | CCGTACACCTTCCCGCAGCC |
| ***bglap*** | NM_001083857.3 | GCCTGATGACTGTGTGTCTGAGCG | AGTTCCAGCCCTCTTCTGTCTCAT |
| ***sost*** | XM_021480342.1 | ACAATGAATCGGGCGAAGAA | GTTCTGAGGCTCCATAAGTCC |
| ***ccnd1*** | NM_131025 | CCAACTTCCTCTCGCAAGTC | TGGTCTCTGTGGAGATGTGC |
| ***axin2*** | NM_131561 | ACCCTCGGACACTTCAAGGAA | TCACTGGCCCTTTTGAAGAAGTAT |

| ***rplp0*** | NM_131580.2 | CTGAACATCTCGCCCTTCTC | TAGCCGATCTGCAGACACAC |
| --- | --- | --- | --- |

| ***rpl13a*** | NM_212784.1 | TCTGGAGGACTGTAAGAGGTATGC | AGACGCACAATCTTGAGAGCAG |
| --- | --- | --- | --- |

**Supplementary Figure S3.** Schematic diagram summarizing all the upregulated and downregulated genes in the probiotic treated fins at 5 DPA and 10 DPA.
